# Elemental Metabolomics: Modulation of Egg Metallome with Flavonoids, an Exploratory Study

**DOI:** 10.3390/antiox8090361

**Published:** 2019-09-01

**Authors:** Athanasios C. Pappas, Evangelos Zoidis, Michael Goliomytis, Panagiotis E. Simitzis, Kyriaki Sotirakoglou, Maria A. Charismiadou, Christos Nikitas, George Danezis, Stelios G. Deligeorgis, Constantinos A. Georgiou

**Affiliations:** 1 Laboratory of Nutritional Physiology and Feeding, Department of Animal Science, Agricultural University of Athens, 11855 Athens, Greece; 2 Laboratory of Animal Breeding and Husbandry, Department of Animal Science, Agricultural University of Athens, 11855 Athens, Greece; 3 Laboratory of Plant Breeding and Biometry, Department of Crop Science, Agricultural University of Athens, 11855 Athens, Greece; 4 Chemistry Laboratory, Department of Food Science and Human Nutrition, Agricultural University of Athens, 11855 Athens, Greece

**Keywords:** egg, flavonoids, hesperidin, metallome, naringin, vitamin E

## Abstract

The basic principles of elemental metabolomics were applied to investigate whether alteration of egg metallome could be achieved after two flavonoids addition, namely hesperidin and naringin in diets of laying hens. A total of 72 hens were divided into six groups: Control (C) (basal diet), E1 (750 mg hesperidin/kg diet), E2 (1500 mg hesperidin/kg diet), N1 (750 mg naringin/kg diet), N2 (1500 mg naringin/kg diet), and VE (200 mg vitamin E/kg diet). The same diet was provided to birds of all treatments, with the exception of added supplements. The diets had the same vitamin and mineral premix; thus, all birds received the same number of elements because no differences on feed intake existed. The egg elemental profile consisted of As, Ca, Cd, Co, Cr, Cu, Fe, Mg, Mn, Mo, Ni, Pb, Sb, Se, Sr, V, Zn, and was determined using ICP-MS. Flavonoid supplementation altered the elemental profile. Most notably, in both albumen and yolk, hesperidin increased Ni, Pb, and Sr concentration while it decreased that of Co and Sb. Naringin increased Cd, Cr, Cu, Ni, and V and lowered the concentration of Co and Sb in both yolk and albumen. Vitamin E supplementation, in comparison to the control, decreased Co in both albumen and yolk and also raised Sb in albumen. Flavonoid presence led to the differences in deposition of certain trace minerals in egg compared to that of hens fed a basal diet or a diet with vitamin E supplementation.

## 1. Introduction 

Biometals are considered the trace metals that play a role in the normal body function [1]. Metallome is defined as the entirety of metal- and metalloid species that are present in a biological system [2]. Inside the body, several elements interact and compete with each other during absorption and homeostasis. Elemental metabolomics focuses on quantification and biomonitoring of elements in biological samples. Recently, the role of elemental metabolomics as an applicable methodology in nutrition, food science, and agriculture has emerged [3,4]. Elemental metabolomics basic principles incorporate, but are not restricted to, proper sample preparation for elemental analysis, use of standard reference materials during inductively coupled plasma mass spectroscopy (ICP-MS) analysis, and proper handling of data, statistical analysis, and reporting [4].

Consumer demand for food products of superior quality led to functional foods and designer eggs [5] that include but are not limited to omega-3 eggs, eggs with lower cholesterol, eggs enriched in vitamin E, pigments, and minerals [6,7,8,9]. Selenium- and iodine-enriched eggs represent some of the most commonly available mineral-enriched eggs [10,11]. Dietary enrichment is achieved by the addition of a mineral complex to layer diet, either in organic or inorganic form [12].

Flavonoids constitute a class of compounds derived by plants, present in fruits and vegetables [13]. They are involved in the antioxidant defense system because they have metal chelating and free radical scavenging properties [14,15]. They share a common phenyl-benzopyrone skeleton and any differences between them are because of the positions and numbers of hydroxyl or methoxylated functional groups, as well as the inter-saccharide linkage and the number and position of different saccharides involved in glycosylation [16].

Naringin has the molecular formula C_27_H_32_O_14_ [17] and hesperidin has the following molecular formula C_28_H_34_O_15_ [18]. Naringin is mainly found in grapefruits and other citrus fruits [19] and has been associated with reduction of cholesterol level [20] and improvement of antioxidant activity [21,22]. Currently, naringin is listed in the EU register of flavoring substances, permitting unrestricted usage in food [23]. Hesperidin is found in citrus fruits and has been linked to improved oxidative stability of eggs and meat [22] as well as to mitigate the toxic effects of heavy metals [24]. In 2010, European Food Safety Authority (EFSA) [25] evaluated naringin and the hesperetin, the aglycone of hesperidin, as flavoring compounds to be applied to the commerce materials, and concluded that these flavonoids are metabolized to innocuous products that do not cause any safety concerns [25]. Furthermore, EFSA considered it safe for the consumer to use naringin in animal nutrition and stated that the use of naringin in animal nutrition does not pose a danger for the environment [23].

Several studies investigated the interactions between flavonoids and specific elements on laboratory animals and reported varying results depending on the polyphenols used and the elements examined. In detail, supranutritional doses of silibinin (100 mg/kg), epigallocatechin gallate (25 mg/kg), quercetin (50 mg/kg), and rutin (500 mg/kg) were added to rat nutrition and reported increased absorption of zinc, copper, and iron and their availability in brain, kidney, and liver [26]. Similarly, the extent to which different polyphenols (chlorogenic acid, caffeic acid, catechin, and rutin) influence zinc and copper absorption in rats was investigated by Coudray et al. [27] and reported that polyphenols may decrease non-hem iron absorption and negatively affect zinc absorption in the rat. Furthermore, a reducing effect of tannic acid on iron but not on zinc, copper, and manganese of rats was observed [28]. The relation of flavonoids to selected elements was also studied in vitro in red blood cells [29] and Caco-2 cells [30].

The aforementioned studies reveal that flavonoid compounds interact with specific minerals on metabolism, with potential implications from a nutritional point of view on humans and animals. Given that studies on flavonoids and minerals on productive animals are sparse, the present study was part of a project and was designed to assess the effects of dietary supplementation of antioxidants on animal product quality. Previously, it was evaluated how dietary supplementation with natural citrus flavonoids, naringin and hesperidin, affected laying performance of hens, yolk oxidative stability, level of cholesterol, egg quality, and cellular immunity parameters [31]. The aim of the present study was to examine whether the use of two flavonoids, namely naringin and hesperidin, can affect the deposition of minerals on egg, which is a staple food of great importance for human nutrition.

## 2. Materials and Methods

### 2.1. Study Design, Sample Selection, and Preparation

The methods used in the present study followed the guidelines of the directive 2010/63/EU of the European Parliament and of the Council on the protection of animals used for scientific purposes and were approved by the Research Ethics Committee of the Faculty of Animal Science of the Agricultural University of Athens (Ethical protocol code: 21-26092013). During the whole trial, chickens were handled in compliance with national and EU regulations and laws and in accordance with the guidelines and principles for the care of animals in experimentation.

Details of the genotype, nutrition, and husbandry of the birds used in this study have been described previously [31]. In brief, 72 brown (Brown-Classic) Lohmann laying hens were randomly assigned into 6 equal treatment groups (12 hens each). The birds were individually caged, and the dimensions of each cage were 0.5 m wide, 0.5 m deep, and 0.5 m high at the front and 0.43 m high at the rear. One of the groups served as a control (C) and was given a commercial basal diet without any added supplement, whereas the other five groups were given the same diet, further supplemented with hesperidin (Sigma-Aldrich, Co., USA) at 750 mg/kg (Ε1) or at 1500 mg/kg (Ε2), naringin (Alfa Aesar GmbH & Co KG, Germany) at 750 mg/kg (N1) or at 1500 mg/kg (N2), or α-tocopheryl acetate (DSM Nutritional Products, Greece) at 200 mg/kg (VE). The decision for selection of the aforementioned inclusion concentrations was based on previous findings from our research team [22,32]. The duration of the study was 9 weeks. Water was provided ad libitum throughout the experimental period and the light regimen was 16 h of continuous light per day. The control diet consisted of a commercial concentrate mixture containing maize (54%), soybean meal (24.6%), wheat (5%), wheat bran (4.6%), soybean oil (1.2%), limestone (8.9%), monocalcium phosphate (0.9%), sodium chloride (0.24%), sodium bicarbonate (0.27%), methionine (0.11%), choline (0.06%), phytase (0.006%), antioxidant (Ethoxyquin, 0.01%), pigment (Carophyll Red©, 0.004%), and a vitamin and mineral premix (0.1%). The vitamin and mineral premix was identical for all treatments and provided per kg of diet 10.000 I.U retinol, 2.500 I.U cholecalciferol, 30 mg tocopherol, 5 mg menadione, 1 mg thiamin, 5 mg riboflavin, 3 mg pyridoxine, 0.020 mg cyanocobalamin, 30 mg nicotinic acid, 10 mg pantothenic acid, 0.8 mg folic acid, 0.10 mg biotin, 10 mg ascorbic acid, 450 mg choline chloride, 0.5 mg iodine (K-iodide), 25 mg iron (Fe(II)-sulphate), 120 mg manganese (Mn(II)-oxide), 100 mg zinc (Zn-oxide), 10 mg copper (CuSO_4_.5H_2_O), 0.2 mg cobalt (Co(II)-carbonate), and 0.3 mg selenium (Na-selenite). Metabolizable energy and crude protein were 11.48 MJ and 175 g per kg feed, respectively. The same diet was provided to all birds of treatments, with the exception of added supplements. The diets had the same vitamin and mineral premix; thus, all birds received the same number of elements because no differences on feed intake were noted [31]. At the 9th week, eggs randomly collected from 8 hens per dietary group were subjected to trace and macro-element analysis.

### 2.2. Determination of Egg Trace and Macro Elements

The trace and macro element analysis included 17 elements, namely: As, Ca, Cd, Co, Cr, Cu, Fe, Mg, Mn, Mo, Ni, Pb, Sb, Se, Sr, V, and Zn. In detail, the elemental profile included elements labelled as major structural components (Ca), responsible for activation or signaling (Ca, Mg), components of enzymes or hormones (Co, Cr, Cu, Fe, Mn, Mo, Ni, Se, V, Zn), toxic (As, Cd, Pb), and others without clear defined function (Sb, Sr). Inductively coupled plasma mass spectrometry, ICP-MS (Perkin Elmer, Elan 9000, Perkin Elmer Life and Analytical Sciences Inc, Waltham, MA, USA) was used for analysis as described previously [33,34].

In brief, the instrumental parameters of the equipment used were as follows: Nebulizer flow 0.75 L/min, lens voltage 7 V, ICP RF power 950 W, pulse stage voltage 950 V. Initially, a complete digestion of the samples was performed with a microwave digestion system (CEM, Mars X-Press, Matthews, NC, USA). Then, 10 ml of concentrated HNO_3_ (65% w/v, Suprapur, Merck, Darmstadt, Germany) were added in samples (1 g wet weight) of albumen or yolk. The samples were heated in the microwave-accelerated digestion system according to the following program: The power was ramped during 20 min from 100 W to 1200 W and held for 15 min. The temperature reached a maximum of 200 °C and was followed by a cool down cycle for 15 min. Losses of volatile element compounds did not occur as the tubes were sealed during heating. The samples were then filtered with disposable syringe filters (Chromafil, Macherey-Nagel, Duren, Germany) and diluted 50-fold with reversed osmosis water (Milli-Q Water Purification Systems, Billerica, MA, USA) prior to injection in the ICP-MS instrument. Standard solutions used for calibration curves were prepared from high purity standards (Inorganic Ventures, Christiansburg, NJ, USA). In order to assess the accuracy of the process, the following standard reference materials were used: NIST-RM 8414—bovine muscle powder (National Institute of Standards & Technology, Gaithersburg, MD, USA), NIST-RM 8415 whole egg powder (National Institute of Standards & Technology, USA), ERM-BB 186 pig kidney (European Commission, Joint Research Centre, institute for reference materials and measurements IRMM, Geel, Belgium).

### 2.3. Statistical Analysis

Data were analyzed using the Statgraphics Centurion statistical package (version 16.1) and are presented as means ± pooled standard errors. Treatment effects on element concentrations and on egg weight and egg component weights were explored using one-way analysis of variance (ANOVA) followed by Duncan’s multiple range test. Kolmogorov–Smirnov test revealed that all variables followed the normal distribution. Principal component analysis (PCA) was also applied in order to decrease the dimensionality of the data and investigate the relationships between the trace and micro elements. Moreover, discriminant analysis was applied to pooled albumen or yolk data to establish those elements capable of distinguishing and classifying samples among the six treatments. Wilk’s lambda (λ) criterion was used for selecting discriminant variables. For all tests, a *p*-value of less than 0.05 was considered to be statistically significant.

## 3. Results 

### 3.1. Concentration of Egg Elements

Concentrations of elements in albumen are presented in Table 1. Most notably, hesperidin addition at a concentration of 750 mg/kg raised Ni (2.3-fold) and Zn (1.5-fold) concentration and lessened that of Co (8.7-fold) compared to control treatment. Higher concentrations of hesperidin (1500 mg/kg) significantly decreased the concentration of Co (10-fold) and Sb (177-fold) and increased that of Pb (1.6-fold) and Sr (35%) compared to control (Table 1). Feed supplementation with naringin at 750 mg/kg, increased, compared to control, the concentration of Cd (13%), Cu (2.1-fold), Ni (3.5-fold), and lessened the concentrations of Ca (80%), Co (7.2-fold), Mn (2-fold), and Sb (245-fold). Naringin addition at 1500 mg/kg lowered, in comparison to the control, the concentration of Ca (1.9-fold), Co (6.1-fold), Mn (3-fold), and Sb (96-fold) and increased Cd (29%), Cr (30%), Cu (3.8-fold), Ni (3.7-fold), and V (10%). Vitamin E supplementation at 200 mg per kg diet, compared to the control, decreased Co concentration (3-fold) and increased that of Sb (1.7-fold) (Table 1). 

Concentrations of elements in egg yolk are shown in Table 2. Addition of 750 mg hesperidin per kg of diet markedly raised the concentration, compared to control, of the following elements: Ca (19%), Cd (23%), Cr (30%), Cu (34%), Fe (22%), Mg (14%), Ni (85%), Pb (84%), Sr (24%), and Zn (27%). Supplementation of 1500 mg/kg hesperidin, compared to control, elevated only the concentration of Pb (83%). At this concentration of supplementation, hesperidin decreased the concentration of Co (2.7-fold) and Sb (57-fold). Naringin added to the diet at 750 mg/kg increased, compared to control, concentrations of four elements, namely Ca (14%), Cr (28%), Fe (18%), and Ni (2-fold) while it decreased Co (3.8-fold) and Sb (221-fold). Addition of 1500 mg/kg naringin decreased the concentration of Sb (70-fold) and Co (3-fold) and positively altered Cd (29%), Cr (33%), Cu (64%), Ni (2.6-fold), and V (13%) concentrations in comparison to the control. Compared to control, feed supplementation with vitamin E at 200 mg per kg diet lessened Co (2.4-fold) concentration (Table 2). 

### 3.2. Principal Components Analysis

**Figure 1:**
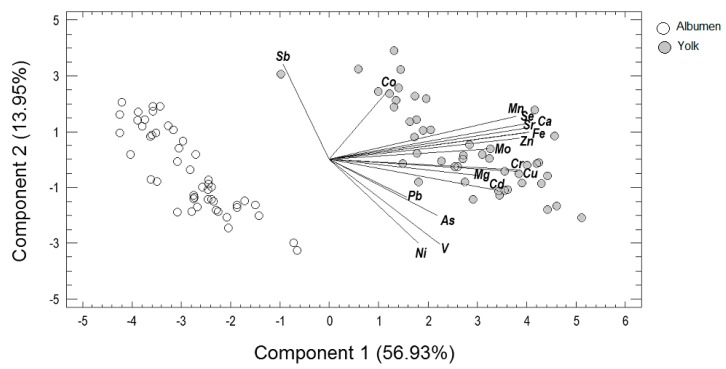
Principal components analysis. Loadings of trace and micro elements for the two first principal components and score plot of albumen or yolk samples.

The concentrations of the 17 analyzed elements in egg yolk and albumen were subjected to principal components analysis (PCA) in order to decrease the dimensionality of the data, investigate the relationships between the trace and micro elements, and detect those elements capable of distinguishing samples. The PCA resulted in three principal components, with eigen values greater than 1.0, a typical statistical cut-off point. The three selected principal components (PCs) accounted for 78.14% of the total variability. Trace and macro elements for the two first PCs and score plot of albumen and yolk samples are presented in Figure 1. In detail, 56.93% of the total variability is explained by PC1, which was defined by the elements Ca, Cd, Cr, Cu, Fe, Mn, Mo, Se, Sr, and Zn. The aforementioned elements were placed away from the axis origin and closed together, indicating a strong positive correlation well represented by PC1. Samples collected from egg yolk were clustered near to these elements and, therefore, egg yolk samples had higher contents of these elements compared to the samples from egg albumen, which were clustered on the negative side of PC1. Thus, a very clear separation of albumen and yolk samples was observed. The second principal component explained another 13.95% of the total variability and was defined by Sb, Ni, and Pb. Ni and Pb were placed closed together on the negative side of PC2, indicating a positive correlation. In contrast, these elements were lying opposite to Sb and, thus, they were found to be negatively correlated with Sb. The third principal component explained another 7.26% of the total variability and it was defined by As, Co, Mg, and V. Samples collected from egg yolk were also clustered near to As, Co, Mg, and V, indicating higher contents of these elements compared to the samples from egg albumen.

### 3.3. Discriminant Analysis

**Figure 2:**
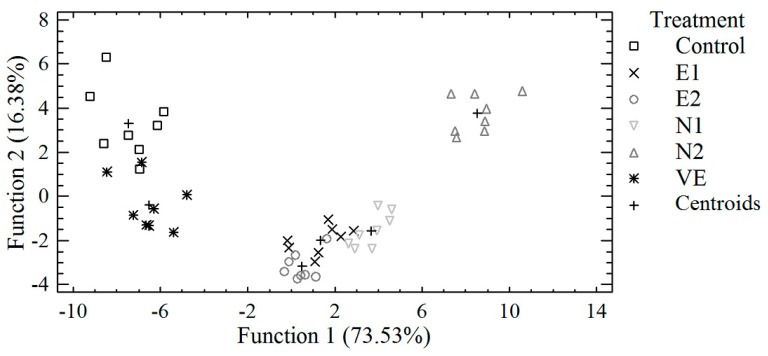
Discriminant plot separating egg albumen samples, according to the dietary treatment, based on trace and macro elements.

**Figure 3:**
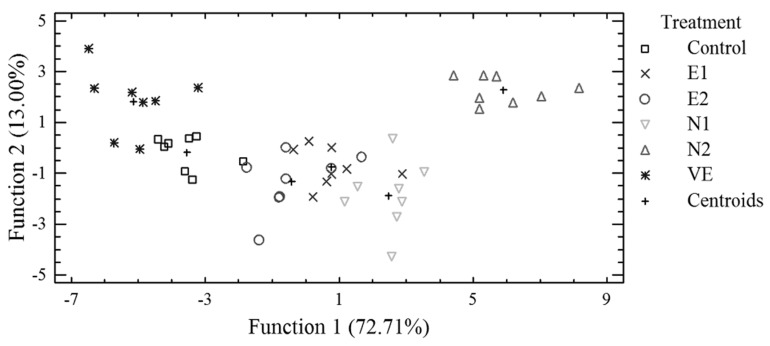
Discriminant plot separating egg yolk samples, according to the dietary treatment, based on trace and macro elements.

A discriminant analysis was further applied to pooled data from egg albumen and/or egg yolk in order to investigate if the samples can be distinguished according to the six dietary treatments, based on selected trace and macro elements concentrations, egg weight, albumen, and/or yolk weight. A discriminant plot from pooled data of albumen is presented in Figure 2. Samples were adequately clustered by the dietary treatment despite some observed overlapping. For distinguishing the samples, analysis revealed that three discriminant functions were statistically significant (*p* < 0.001). Ninety-six percent (96%) of the samples were classified into the correct group according to the dietary treatment. Α stepwise discriminant analysis showed that Ca, Co, Cu, Fe, Mn, and Sb were primarily responsible for the separation of egg albumen samples, according to the dietary treatment. In Figure 3, a discriminant plot separating egg yolk samples, according to the dietary treatment, based on trace and macro elements, is presented. Control samples were clustered near samples with vitamin E supplementation, as in the discriminant plot of albumen and samples from treatments E1, E2, and N1 were also placed close together, indicating similar characteristics. Samples from N2 treatment were clustered in both plots (Figure 2 and Figure 3) away from the other samples and opposite to control and VE samples, indicating different concentrations of the analyzed elements. Three discriminant functions were statistically significant (*p* < 0.01) for distinguishing the samples, leading to a 92% correct classification. A stepwise discriminant analysis revealed that Ni, Sb, Co, Ca, Cu, Sr, and Fe contributed the most to distinguishing the observations.

## 4. Discussion

### 4.1. Flavonoids in Poultry Nutrition

In the present work, higher concentrations for the majority of elements were observed in yolk compared to albumen, indicating that primary elements are deposited into the yolk where most minerals in the egg are stored and from where they can be transferred effectively through the yolk sac to the developing embryo. Elements deposited in the albumen may also be available during embryogenesis [35,36].

An in vitro study that investigated the role of citrus flavonoid in the bioavailability of Ca, Mg, P, Fe, and Zn of eggshells and on the role of hesperidin in inhibition of bone loss revealed that presence of citrus flavonoids may help to increase the concentration of several minerals [37]. In detail, that in vitro study showed that the concentrations of elements that are released from the eggshell is enhanced by the presence of citric acid (Ca, Mg, P, and Zn), ascorbic acid (Fe), or hesperidin (Ca, Fe, Mg, P, and Zn) [37]. Mineral homeostasis is of key importance to maintain a balance between absorption and excretion of these metals and the primary role in this balance is attributed to transport proteins like Mg channels and Zn and Cu transporters [38]. In mammals, protection against Cd toxicity and homeostasis of certain essential metals is attributed to metallothioneins—low molecular weight, cysteine-rich, intracellular proteins—that exhibit high affinity for both essential and non-essential metals [39,40].

Recently, it was reported that hesperetin interacts with lysozyme, an abundant protein in hen egg albumen, inducing structural changes as confirmed from synchronous fluorescence, three-dimensional fluorescence, and circular dichroism analyses [41]. In a review on egg as an antioxidant, it was reported that several proteins of egg albumen may possess metal binding activities and exert antioxidant properties [9]. Ovalbumin exerts antioxidant properties because free thiol (SH) groups in ovalbumin can regulate the redox status and bind metal ions [42]. In turn, ovotransferrin represents 12% of the egg albumen proteins, and as a member of the transferrin family has binding properties with a preference for iron [43].

Both the gastrointestinal tract and the liver play an important role in metabolism of polyphenols [44,45]. After hydrolysis, naringin is absorbed in a similar manner to naringenin and is transformed to common urinary and biliary metabolites [46].

### 4.2. Alteration of Egg Metallome

In the present work, supplementation of layers diet with hesperidin and naringin increased the concentration of Ca, Cd, Cr, Cu, Fe, Mg, Ni, Pb, Sr, V, and Zn in either yolk or albumen compared to control or vitamin E-supplemented fed layers, creating a different elemental profile. Flavonoids affected homeostasis of trace elements in hens as reflected by changes of the elemental profile of egg. Effects of flavonoids on trace element homeostasis may be related to creation of complexes of flavonoids with elements. Taking into account that metal ions are essential for most forms of life because several proteins require a metal as a cofactor [47] and that metal ions have an effect on the hydrogen atom transferring ability of the complex, it is revealed that complexes deactivate oxidants via hydrogen atom transfer [48]; thus, metal chelation is an antioxidant mechanism of flavonoids. Three domains able to react with metal ions can be found in flavonoids, namely the 3′,4′-dihydroxy system placed on the B ring, the 3-hydroxy or 5-hydroxy groups, and the 4-carbonyl group in the C ring [14]. The results of the present study are in line with previous studies on rats reporting that tissue availability of the several elements may be related to the fact that absorption and membrane transport of some metals ions can be enhanced with the formation of complexes and chelates with organic ligands [26].

It is well established that vitamin E and ascorbic acid are among the most effective antioxidants in biologic systems in preventing oxidative detrimental effects [49], despite some randomized controlled trials that revealed that vitamin E has no effect in cardiovascular or cancer prevention [50]. In the present study, two different added flavonoids, namely hesperidin and naringin, and vitamin E supplementation were examined and it was observed that deposition of certain elements was higher in eggs from layers fed flavonoid compared to layers fed supplemented vitamin E, possibly attributed to the chelating ability of flavonoids. Formation of some of these complexes has been reported previously in studies with plants, animal models, or humans. Most notably, in a study with postmenopausal women, Ca supplementation in combination with hesperidin improved bone Ca retention by 5.5% [51]. The authors attributed hesperidin results on the effects on cell signaling pathways that regulate the responsible cells for bone resorption and formation [52]. Furthermore, in obese rats fed a high-carbohydrate or high-fat diet, it was observed that naringin addition exerted a vasodilatation effect [53]. Under this context, naringenin, the aglycone of naringin, expressed a vasodilatation effect in rat arteries, possibly by activating conductance Ca^2+^-activated K^+^ currents in a concentration-dependent way [54]. 

Flavonoids have been shown to display strong antioxidant activity and chelation of iron by providing protection against induced oxidation. Ferrali et al. [55] studied quercetin as an antioxidant against induced oxidation in glutathione-depleted mouse erythrocytes and reported that quercetin chelated iron and penetrated erythrocytes, resulting in noticeable protection against lipid peroxidation and hemolysis. Similarly, Deng et al. [56] examined five flavonoids, namely baicalin, hesperidin, naringin, quercetin, and rutin, and reported that their antioxidant function is chelating iron ions and scavenging peroxyl radicals. Differences between flavonoids may be related to their structure and the chelation site. Mira et al. [57] reported that quercetin has a hydroxyl group in position 3, while luteolin does not possess the 3-hydroxyl group, and naringenin has no double bond between positions 2 and 3 in the C ring. In addition, hesperidin is much more hydrophilic than its aglycone hesperetin, possibly explaining any differences between them [58]. Mira et al. [57] reported that most flavonoids exhibit a yield of redox reactions greater with copper than with iron, given the lower redox potential of Cu^2+^/Cu^+^ in comparison to that of Fe^3+^/Fe^2+^.

In the present work, flavonoids presence resulted in higher concentration of Mg, Zn, and Cd. Previous studies with flavonoids and Mg revealed complexing of flavonoids with this element. In detail, the interaction of quercetin with MgSO_4_·7H_2_O was examined and it was reported that the metal ions significantly alter chemical properties of quercetin [59]. Zhuang et al. [60] induced endothelial cell dysfunction and reported that co-addition of total flavonoids from the *Hippophae rhamnoides* seed residues together with ZnCl_2_ resulted in a chelate complex, which acted as a zinc pool to restore cellular zinc homeostasis. Studies with plants revealed that flavonoids are key factors for the tolerance of cadmium [61].

Eggs fortified in certain elements, such as selenium [62] and iodine [10,63], are already produced through feed supplementation. Suitable organic forms of elements may be used to overcome any bioavailability limitation [64]. Assessing the complete elemental profile, as envisaged by elemental metabolomics [4], provides the potential in creating tailor-made eggs through fine tuning of the elemental supplementation using the whole elemental content and the simultaneous adjustment of flavonoid concentration. Results of the present study point out that there are complex interactions that cannot be attributed just to chelation of elements, and thus, further research is needed. It is postulated that interactions could be resolved through feeding experiments at different concentrations of elemental content and simultaneously different concentrations of flavonoids. 

In the present exploratory study, the flavonoids that were used were in a pure chemical form. It is postulated that any natural agricultural by-product rich in phenolics and flavonoids, like the by-products of olive oil, fruit juice, and the wine industry, can be used. 

## 5. Conclusions

The present study indicated that naringin and hesperidin can be used to alter the elemental profile of egg because the presence of flavonoids was capable of altering the concentration of certain elements in the egg compared to that of hens fed a basal diet or a diet supplemented with vitamin E. Results may be attributed to the chelating ability of flavonoids with metal ions and creation of complex compounds that became absorbed. The present study indicated that in the future, tailor-made eggs for specific health and nutritional needs could be created. Tailor-made eggs may address specific needs such as diets rich in iron [65].

## Figures and Tables

**Table 1:** The effect of dietary supplementation with hesperidin and naringin on trace- and macro-elements concentrations in the albumen of egg (*n* = 8).

Elements (μg/kg)				Treatment				*p*-Value
	C	E1	E2	N1	N2	VE	SEM	
As	4.26	4.39	4.58	4.55	4.56	4.01	0.228	0.457
Ca	178,715 ^b^	170,556 ^b^	209,284 ^b^	100,415 ^a^	95,117 ^a^	162,672 ^b^	16,884.5	<0.001
Cd	2.45 ^a^	2.65 ^a,b^	2.56 ^a,b^	2.76 ^b^	3.15 ^c^	2.41 ^a^	0.072	<0.001
Co	24.95 ^b^	2.85 ^a^	2.50 ^a^	3.46 ^a^	4.09 ^a^	8.15 ^a^	1.642	<0.001
Cr	93.78 ^a^	96.90 ^a^	94.66 ^a^	101.48 ^a,b^	122.85 ^b^	84.63 ^a^	5.273	<0.001
Cu	326.48 ^a^	570.76 ^a,b^	511.90 ^a,b^	698.76 ^b^	1241.14 ^c^	311.84 ^a^	84.667	<0.001
Fe	17,202.9 ^a,b,c^	18,630 ^a,b,c^	18,981 ^b,c^	19,461.3 ^c^	16,256.1 ^a^	16,433.4 ^a,b^	846.16	0.037
Mg	83,029.0	95,438.6	94,701.7	93,771.8	84,495.7	87,567.8	4018.99	0.121
Mn	168.85 ^b^	111.56 ^a,b^	104.18 ^a,b^	82.89 ^a^	55.41 ^a^	112.03 ^a,b^	18.620	0.004
Mo	115.74	147.27	128.29	176.34	134.06	117.04	29.050	0.691
Ni	33.88 ^a^	78.78 ^b^	60.31 ^a,b^	117.51 ^c^	125.11 ^c^	33.83 ^a^	9.150	<0.001
Pb	19.21 ^a^	22.55 ^a,b^	30.84 ^b^	25.0 ^a,b^	25.41 ^a,b^	16.39 ^a^	2.682	0.009
Sb	3.19 ^b^	1.21 ^a,b^	0.018 ^a^	0.013 ^a^	0.033 ^a^	5.52 ^c^	0.546	<0.001
Se	118.68 ^a,b^	110.53 ^a,b^	125.99 ^b^	99.73 ^a,b^	91.69 ^a^	118.98 ^a,b^	7.538	0.022
Sr	116.84 ^a^	120.24 ^a,b^	157.49 ^b^	115.59 ^a^	108.05 ^a^	107.60 ^a^	8.900	0.003
V	103.46 ^a^	106.80 ^a,b^	108.19 ^a,b^	111.09 ^a,b^	113.66 ^b^	101.88 ^a^	2.261	0.005
Zn	3459.2 ^a,b^	5262.5 ^c^	4185.9 ^a,b,c^	5109.2 ^b,c^	3306.8 ^a^	3276.4 ^a^	551.7	0.034

C: no additive, E1 and E2: 750 and 1500 mg hesperidin per kg feed, respectively, N1 and N2: 750 and 1500 mg naringin per kg feed, respectively, VE: 200 mg a-tocopheryl acetate (vitamin E) per kg feed. ^a,b,c^ means in a row sharing no common superscript differ significantly (*p* < 0.05).

**Table 2:** The effect of dietary supplementation with hesperidin and naringin on trace- and macro-elements concentrations in the yolk of egg (*n* = 8).

Elements (μg/kg)				Treatment				*p*-Value
	C	E1	E2	N1	N2	VE	SEM	
As	4.59	5.06	5.19	4.75	5.31	4.38	0.245	0.068
Ca	1.452 × 10^6 a^	1.728 × 10^6 b^	1.592 × 10^6 a,b^	1.653 × 10^6 b^	1.592 × 10^6 a,b^	1.402 × 10^6 a^	47576.6	<0.001
Cd	3.06 ^a^	3.78 ^b^	3.35 ^a,b^	3.35 ^a,b^	3.94 ^b^	2.90 ^a^	0.165	<0.001
Co	30.44 ^b^	18.43 ^a,b^	11.21 ^a^	7.98 ^a^	9.85 ^a^	12.44 ^a^	3.869	0.002
Cr	144.70 ^a^	188.11 ^b^	160.81 ^a,b^	185.79 ^b^	192.93 ^b^	131.88 ^a^	8.148	<0.001
Cu	1485.97 ^a^	1993.15 ^b^	1755.32 ^a,b^	1883.93 ^a,b^	2444.86 ^c^	1477.16 ^a^	105.60	<0.001
Fe	76,446.9 ^a^	93,753.8 ^b^	83,928.6 ^a,b^	90,547.8 ^b^	86,095.7 ^a,b^	76,851.9 ^a^	3690.15	0.008
Mg	98,988.3 ^a^	112,877.1 ^b^	106,972.2 ^a,b^	107,764.1 ^a,b^	102,809.3 ^a,b^	95,459.3 ^a^	2675.86	<0.001
Mn	1132.21	1222.97	1093.19	1207.33	1173.03	1193.88	103.26	0.949
Mo	843.21	1509.76	832.99	1203.24	1360.28	735.24	255.92	0.189
Ni	56.41 ^a,b^	104.49 ^c^	86.83 ^b,c^	116.43 ^cd^	145.68 ^d^	52.91 ^a^	7.845	<0.001
Pb	20.85 ^a^	38.40 ^b^	38.21 ^b^	23.30 ^a,b^	27.84 ^a,b^	21.41 ^a^	4.984	0.036
Sb	2.88 ^b,c^	1.13 ^a,b^	0.05 ^a^	0.013 ^a^	0.041 ^a^	4.45 ^c^	0.483	<0.001
Se	457.38	520.91	466.48	483.38	472.84	466.58	19.247	0.245
Sr	534.76 ^a^	665.54 ^b^	584.34 ^a,b^	600.43 ^a,b^	585.64 ^a,b^	525.85 ^a^	28.405	0.017
V	105.29 ^a,b^	114.51 ^b,c^	114.33 ^b,c^	114.33 ^b,c^	119.26 ^c^	102.84 ^a^	2.545	<0.001
Zn	43,526.7 ^a^	55,172.8 ^b^	48,275.0 ^a,b^	48,697.4 ^a,b^	47,815.2 ^a,b^	44,298.0 ^a^	2119.24	0.006

C: no additive, E1 and E2: 750 and 1500 mg hesperidin per kg feed, respectively, N1 and N2: 750 and 1500 mg naringin per kg feed, respectively, VE: 200 mg a-tocopheryl acetate (vitamin E) per kg feed. ^a,b,c,d^ means in a row sharing no common superscript differ significantly (*p* < 0.05).
